# Diagnostic value of saline infusion test–derived biochemical parameters for primary aldosteronism subtyping

**DOI:** 10.3389/fendo.2026.1923356

**Published:** 2026-10-06

**Authors:** Yusuf Öztürk, Feridun Karakurt

**Affiliations:** 1 Department of Endocrinology and Metabolism, Sakarya University Training and Research Hospital, Sakarya, Türkiye; 2 Department of Endocrinology and Metabolism, Necmettin Erbakan University Faculty of Medicine, Konya, Türkiye

**Keywords:** adrenal venous sampling, aldosterone-to-potassium ratio, plasma aldosterone concentration, primary aldosteronism, saline infusion test, suppression index, unilateral primary aldosteronism

## Abstract

**Background:**

Current clinical practice guidelines recommend adrenal venous sampling (AVS) for subtype classification in patients with primary aldosteronism (PA) who are candidates for adrenalectomy; however, AVS may be considered unnecessary in selected young patients with a high probability of unilateral disease. The additional diagnostic information provided by baseline and saline infusion test (SIT)-derived biochemical parameters when evaluated alongside clinical and radiological features remains uncertain. We aimed to compare the diagnostic performance of baseline and SIT-derived biochemical parameters for predicting unilateral PA and to evaluate the additional diagnostic information provided by each parameter when individually added to a common clinical-radiological framework.

**Methods:**

This retrospective study included 73 patients with PA (41 unilateral and 32 bilateral) who underwent successful AVS for subtype classification. Baseline plasma aldosterone concentration (PAC), baseline aldosterone-to-potassium ratio (APR), post-SIT PAC, SIT-derived APR, and the suppression index were evaluated. Diagnostic performance was assessed using receiver operating characteristic (ROC) curve analysis, while their associations within a common clinical-radiological model were evaluated using multivariable logistic regression analysis.

**Results:**

Post-SIT PAC and SIT-derived APR showed the highest numerical discriminative performance among the evaluated biochemical parameters for predicting unilateral PA (AUC = 0.817 and 0.837, respectively). Adding either parameter individually to the common clinical-radiological model significantly improved model fit (likelihood-ratio *p* = 0.014 and *p* = 0.028, respectively); however, the increases in AUC were not statistically significant (ΔAUC = 0.022, *p* = 0.185 and ΔAUC = 0.017, *p* = 0.107, respectively). Post-SIT PAC and SIT-derived APR were strongly correlated (Spearman’s ρ = 0.983, *p* < 0.001), and their ROC-derived thresholds yielded concordant classifications in 97.3% of patients.

**Conclusion:**

Our findings suggest that post-SIT PAC shows useful discriminative performance for predicting unilateral PA and may contribute to subtype assessment when considered alongside clinical and radiological features. These findings require validation in independent cohorts.

## 1 Introduction

Primary aldosteronism (PA) is one of the leading causes of secondary hypertension and is largely amenable to targeted treatment (1, 2). Its unilateral and bilateral subtypes require fundamentally different therapeutic approaches. While unilateral PA can be effectively treated with adrenalectomy, resulting in biochemical remission and substantial clinical improvement, bilateral PA is primarily managed with long-term mineralocorticoid receptor antagonist therapy (3). Therefore, accurate subtype classification is essential not only for selecting the appropriate treatment strategy but also for reducing the long-term cardiovascular and renal complications associated with chronic aldosterone excess (4–6).

Adrenal venous sampling (AVS) is currently regarded as the reference standard for distinguishing unilateral from bilateral PA (7). However, its widespread use is limited by its invasive nature, technical complexity, dependence on experienced operators, and limited availability in many centers (8). Current clinical practice guidelines recommend AVS for subtype classification in patients with PA who are candidates for adrenalectomy; however, AVS may be considered unnecessary in selected young patients in whom the probability of unilateral PA is very high. This phenotype is characterized by features such as young age, spontaneous hypokalemia, marked PA, and a unilateral adrenal adenoma on computed tomography (CT) (9). Although the guideline exemplifies “marked PA” by biochemical features such as spontaneous hypokalemia, elevated plasma aldosterone concentration (PAC), and suppressed renin, the relative contribution of different biochemical parameters to the prediction of unilateral disease remains uncertain.

In recent years, biochemical parameters derived from the saline infusion test (SIT) have attracted increasing interest as potential predictors of PA subtypes. Several studies have reported promising diagnostic performance for post-SIT plasma aldosterone concentration (PAC), the aldosterone suppression rate, and the aldosterone-to-potassium ratio (APR) in predicting unilateral disease (10–12). Whereas baseline biochemical parameters such as PAC and APR reflect the biochemical phenotype of PA under baseline conditions, parameters obtained after SIT may provide dynamic information on the persistence of aldosterone secretion despite standardized sodium and volume loading. SIT-derived parameters may therefore provide information that is distinct from or complementary to baseline biochemical measurements for identifying patients with a high probability of unilateral PA. However, most previous studies have focused on the individual diagnostic performance of these biomarkers, and the comparative contributions of baseline and SIT-derived biochemical parameters within the same clinical-radiological framework have not been adequately evaluated.

In the present study, we evaluated the diagnostic performance of baseline biochemical parameters, SIT-derived biochemical parameters and indices, and radiological findings for predicting unilateral PA in patients with AVS-confirmed subtype classification. In addition, we comparatively investigated the additional diagnostic information provided by baseline and SIT-derived biochemical parameters when individually added to a common clinical-radiological framework consisting of age, hypokalemia, and unilateral adrenal findings on CT.

## 2 Methods

### 2.1 Study design and patient selection

This retrospective, single-center observational study was designed to evaluate the diagnostic value of clinical, biochemical, radiological, and SIT-derived parameters for predicting unilateral disease in patients with PA. The study was approved by the Non-Pharmaceutical and Non-Medical Device Research Ethics Committee of Necmettin Erbakan University (Approval No. 2025/5450; January 10, 2025) and was conducted in accordance with the principles of the Declaration of Helsinki. Patients evaluated for PA at the Division of Endocrinology and Metabolism, Faculty of Medicine, Necmettin Erbakan University, between January 2020 and December 2024 were retrospectively screened (**Figure 1**).

**Figure 1:**
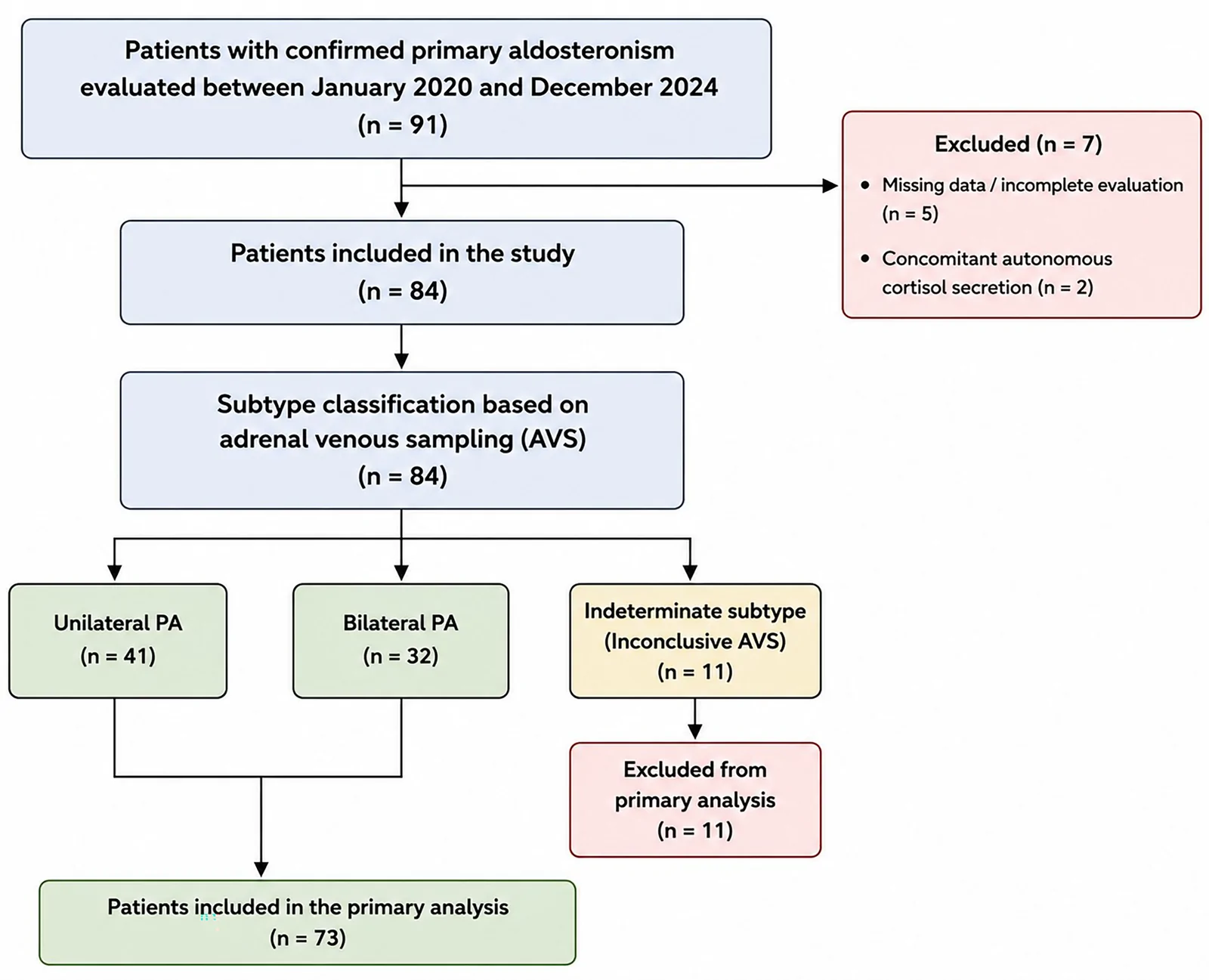
Flow diagram of patient selection and study cohort formation. Patients with confirmed primary aldosteronism evaluated between January 2020 and December 2024 were screened for eligibility. After excluding patients with incomplete data or concomitant autonomous cortisol secretion, 84 patients underwent adrenal venous sampling (AVS). Patients with indeterminate AVS results were excluded from the primary analysis. The final study cohort consisted of 41 patients with unilateral primary aldosteronism and 32 patients with bilateral primary aldosteronism (n = 73).

During the study period, 91 patients with confirmed PA were assessed for eligibility. Five patients were excluded because of incomplete clinical data or an incomplete diagnostic evaluation, and two were excluded because of concomitant autonomous cortisol secretion. Consequently, the study cohort consisted of 84 patients.

Patients were classified according to AVS findings as unilateral PA, bilateral PA, or indeterminate subtype. Based on AVS results, 41 patients were diagnosed with unilateral PA, 32 with bilateral PA, in 11 patients, successful bilateral adrenal vein cannulation could not be achieved; therefore, AVS was considered technically unsuccessful and these patients were classified as having an indeterminate subtype. Patients with an indeterminate subtype were excluded from the primary analysis. Accordingly, the final study population comprised 73 patients with AVS-confirmed subtype classification, including 41 patients with unilateral PA and 32 with bilateral PA.

Among the 41 patients with unilateral PA, 33 underwent adrenalectomy. Histopathological examination of the surgical specimens demonstrated adenoma in 30 patients and adrenal cortical hyperplasia in three patients. None of the patients with bilateral PA underwent adrenalectomy.

Demographic and clinical characteristics, including age, sex, duration of hypertension, number of antihypertensive medications, comorbidities, biochemical findings, SIT results, and adrenal CT findings, were retrieved from the institutional electronic medical records. The following variables were included in the analyses: baseline PAC, plasma renin activity (PRA), post-SIT PAC, aldosterone-to-renin ratio (ARR), baseline APR, SIT-derived APR, and adrenal adenoma diameter. For the purposes of this study, APR was defined as PAC divided by the lowest serum potassium concentration recorded during the diagnostic evaluation before potassium replacement or other therapeutic intervention. Accordingly, baseline APR was calculated using baseline PAC, whereas SIT-derived APR was calculated using post-SIT PAC; the same pre-intervention minimum serum potassium concentration was used as the denominator for both ratios. The suppression index was calculated as the ratio of post-SIT PAC to baseline PAC (post-SIT PAC/baseline PAC).

### 2.2 Diagnosis of primary aldosteronism and subtype classification

The diagnostic evaluation of primary aldosteronism (PA) was performed in accordance with the 2016 Endocrine Society Clinical Practice Guideline (7). Patients with suspected PA initially underwent measurement of plasma aldosterone concentration (PAC), plasma renin activity (PRA), and the aldosterone-to-renin ratio (ARR). Plasma aldosterone concentration was measured using an immunoassay, whereas plasma renin activity was determined by radioimmunoassay (RIA). In patients with hypokalemia, serum potassium levels were corrected to the normal range before confirmatory testing. Antihypertensive medications known to interfere with ARR were discontinued whenever clinically feasible or replaced with alternative agents recommended by the guideline before confirmatory testing (7).

Patients with a positive screening test subsequently underwent a saline infusion test (SIT) for biochemical confirmation. All patients were evaluated in the morning after an overnight fast. The SIT was performed using the recumbent (supine) protocol. Patients remained in the supine position for at least one hour before the test and throughout the infusion period. Subsequently, 2 L of 0.9% isotonic saline was infused intravenously over four hours. Plasma aldosterone concentration was measured immediately before and after completion of the infusion. The diagnosis of PA was confirmed according to the diagnostic criteria recommended by the 2016 Endocrine Society Clinical Practice Guideline (7).

All patients with confirmed PA underwent adrenal computed tomography (CT) to evaluate adrenal morphology. Subtype classification was subsequently established by adrenal venous sampling (AVS) performed under continuous adrenocorticotropic hormone (ACTH) stimulation. Successful bilateral adrenal vein cannulation was defined by a selectivity index >5, calculated as the ratio of adrenal vein cortisol to peripheral venous cortisol. The lateralization index was calculated as the ratio of the cortisol-corrected aldosterone concentration (aldosterone/cortisol) in the dominant adrenal vein to that in the contralateral adrenal vein, with a lateralization index >4 considered indicative of unilateral aldosterone secretion (7, 8). Contralateral suppression was also assessed as part of the AVS interpretation. Patients with successful bilateral adrenal vein cannulation and a lateralization index >4 were classified as having unilateral PA, whereas those with successful bilateral cannulation but without lateralization were classified as having bilateral PA. AVS procedures in which successful bilateral adrenal vein cannulation could not be achieved were considered technically unsuccessful; these patients were classified as having an indeterminate subtype and were excluded from the primary analysis.

### 2.3 Statistical analysis

Statistical analyses were performed using IBM SPSS Statistics for Windows, version 22.0 (IBM Corp., Armonk, NY, USA). Data distribution was assessed using the Shapiro–Wilk test together with visual inspection of histograms and Q–Q plots. Normally distributed continuous variables are presented as mean ± standard deviation (SD), whereas non-normally distributed variables are expressed as median (interquartile range [IQR]). Categorical variables are presented as frequencies and percentages.

Comparisons between patients with unilateral and bilateral primary aldosteronism were performed using the independent-samples *t* test or the Mann–Whitney *U* test, as appropriate. Categorical variables were compared using the chi-square test or Fisher’s exact test, as appropriate.

Receiver operating characteristic (ROC) curve analysis was performed to evaluate the discriminatory performance of continuous biochemical and radiological variables for unilateral primary aldosteronism. Areas under the ROC curves (AUCs) with corresponding 95% confidence intervals (CIs) were calculated. Optimal cut-off values were determined using the maximum Youden index and were subsequently used to dichotomize continuous variables. Because these cut-off values were derived and evaluated in the same cohort, they were considered exploratory and cohort-specific.

Univariable logistic regression analysis was performed to identify variables associated with unilateral primary aldosteronism. A clinical-radiological base model consisting of age <35 years, hypokalemia (serum potassium <3.5 mmol/L), and a unilateral adrenal adenoma on computed tomography was established. Biochemical variables were intentionally not incorporated into the base model because the objective was to compare the additional diagnostic information provided by baseline and SIT-derived biochemical parameters when each was individually added to the same clinical-radiological framework. Accordingly, baseline PAC, baseline APR, suppression index, post-SIT PAC, and SIT-derived APR were each entered separately into the base model.

Expanded models were compared with the clinical-radiological base model using likelihood-ratio tests. Differences in discrimination were assessed by the change in AUC (ΔAUC), with corresponding 95% CIs and paired comparisons of AUCs. Internal validation was performed using 2,000 bootstrap resamples to estimate optimism-corrected AUCs. As an additional sensitivity analysis, the entire cut-off selection procedure based on the maximum Youden index was repeated within each bootstrap resample to account for optimism related to data-driven threshold selection. Calibration was assessed using Brier scores, bootstrap-corrected calibration slopes, and calibration plots comparing predicted and observed probabilities.

Classification performance of the clinical-radiological base model and each expanded model was additionally evaluated at a predicted-probability threshold of 0.50. Sensitivity, specificity, positive predictive value (PPV), negative predictive value (NPV), and the numbers of true-positive, false-positive, true-negative, and false-negative classifications were calculated, with unilateral primary aldosteronism considered the positive outcome. Because post-SIT PAC and SIT-derived APR showed highly similar diagnostic performance, their relationship was further evaluated using Spearman’s rank correlation coefficient. Agreement between classifications based on their respective ROC-derived thresholds was assessed using Cohen’s κ coefficient.

Odds ratios (ORs) are presented with 95% CIs. All statistical tests were two-sided, and a *p* value <0.05 was considered statistically significant.

## 3 Results

### 3.1 Clinical, biochemical, and radiological characteristics of the study population

A total of 73 patients with AVS-confirmed primary aldosteronism subtype classification were included in the primary analysis, comprising 41 patients with unilateral PA and 32 with bilateral PA (**Figure 1**). The clinical, biochemical, radiological, and saline infusion test (SIT)-derived characteristics of the study population are summarized in **Table 1**.

**Table 1:** Baseline clinical, biochemical, radiological, and saline infusion test (SIT)-derived characteristics of patients with unilateral and bilateral primary aldosteronism.

Variable	Unilateral PA (n=41)	Bilateral PA (n=32)	P value
Clinical characteristics			
Age, years	46.0 ± 13.5	50.2 ± 10.6	0.154
Age <35 years, n (%)	16 (39.0)	3 (9.4)	**0.004**
Female sex, n (%)	25 (61.0)	17 (53.1)	0.501
Number of antihypertensive medications	3.0 (2.0–3.0)	2.0 (2.0–3.0)	0.673
Comorbidities			
Diabetes mellitus, n (%)	9 (22.0)	11 (34.4)	0.238
Prediabetes, n (%)	3 (7.3)	6 (18.8)	0.140
Coronary artery disease, n (%)	6 (14.6)	4 (12.5)	0.792
Stroke history, n (%)	2 (4.9)	2 (6.3)	0.798
Nephropathy, n (%)	8 (19.5)	7 (21.9)	0.804
Laboratory parameters			
Sodium, mmol/L	142.0 (140.0–143.0)	140.0 (138.0–143.0)	**0.023**
Potassium, mmol/L	3.20 (2.90–3.66)	4.04 (3.48–4.25)	**<0.001**
Hypokalemia (K <3.5 mmol/L), n (%)	26 (63.4)	8 (25.0)	**0.001**
Baseline PAC, ng/dL	38.0 (23.0–50.6)	23.5 (18.9–31.5)	**0.011**
Renin activity, ng/mL/h	0.46 (0.15–1.12)	0.51 (0.24–0.77)	0.991
ARR	57.50 (36.30–230.77)	58.06 (29.57–113.40)	0.331
Post-SIT PAC, ng/dL	31.2 (21.0–51.0)	14.2 (10.1–21.8)	**<0.001**
Derived biochemical indices			
Baseline APR	11.23 (7.13–14.74)	6.19 (4.56–8.90)	**0.001**
SIT-derived APR	10.76 (5.71–15.64)	3.78 (2.36–5.40)	**<0.001**
Suppression index	0.88 (0.67–1.17)	0.61 (0.40–0.81)	**0.002**
Radiological findings			
Unilateral adrenal adenoma on CT, n (%)	32 (78.0)	4 (12.5)	**<0.001**
Adrenal adenoma diameter, mm	17.0 (12.0–22.8)	9.0 (6.0–16.0)	**<0.001**

Data are presented as mean ± standard deviation (SD), median [interquartile range (IQR)], or number (%), as appropriate.

Continuous variables were compared using the Student’s t-test or Mann–Whitney U test, as appropriate. Categorical variables were compared using the χ² test or Fisher’s exact test.

The suppression index was calculated as post-SIT PAC divided by baseline PAC.

APR, aldosterone-to-potassium ratio; ARR, aldosterone-to-renin ratio; CT, computed tomography; IQR, interquartile range; PA, primary aldosteronism; PAC, plasma aldosterone concentration; SD, standard deviation; SIT, saline infusion test.

Bold values indicate statistical significance (p < 0.05).

The proportion of patients younger than 35 years was significantly higher in the unilateral PA group than in the bilateral PA group (*p* = 0.004). Hypokalemia (serum potassium <3.5 mmol/L) was more frequent (*p* = 0.001), and serum potassium concentrations were significantly lower (*p* < 0.001) in patients with unilateral PA. No significant differences were observed between the groups regarding age, sex distribution, number of antihypertensive medications, or comorbidities.

Among the biochemical variables, both baseline PAC and post-SIT PAC were significantly higher in patients with unilateral PA than in those with bilateral PA (*p* = 0.011 and *p* < 0.001, respectively). In contrast, PRA and the ARR did not differ significantly between the two groups.

All SIT-derived biochemical indices were significantly higher in the unilateral PA group. Baseline APR (*p* = 0.001), SIT-derived APR (*p* < 0.001), and the suppression index (*p* = 0.002) were all significantly higher in patients with unilateral PA than in those with bilateral PA.

Radiological evaluation demonstrated that unilateral adrenal adenomas were significantly more frequent in patients with unilateral PA (*p* < 0.001). In addition, adrenal adenoma diameter was significantly greater in the unilateral PA group (*p* < 0.001).

### 3.2 Diagnostic performance analysis

The diagnostic performance of clinical, biochemical, and radiological parameters for predicting unilateral PA is summarized in **Table 2**, and the corresponding ROC curves are presented in **Figure 2**.

**Table 2:** Diagnostic performance of clinical, biochemical, and radiological parameters for predicting unilateral primary aldosteronism.

Variable	AUC (95% CI)	P value	Optimal cut-off value	Sensitivity (%)	Specificity (%)
Clinical and radiological predictors*					
Age <35 years	—	**0.004**	Present	39.0	90.6
Hypokalemia (K <3.5 mmol/L)	—	**0.001**	Present	63.4	75.0
Unilateral adrenal adenoma on CT	—	**<0.001**	Present	78.0	87.5
ROC-derived predictors					
SIT-derived APR	0.837 (0.741–0.933)	**<0.001**	>8.36	63.4	90.6
Post-SIT PAC	0.817 (0.716–0.919)	**<0.001**	>27 ng/dL	63.4	90.6
Adenoma diameter	0.799 (0.668–0.930)	**<0.001**	>11.5 mm	80.0	73.9
Baseline APR	0.789 (0.673–0.905)	**<0.001**	>9.7	65.9	81.2
Baseline PAC	0.730 (0.605–0.856)	**0.002**	>30 ng/dL	63.4	71.9
Suppression index	0.671 (0.531–0.812)	**0.025**	>0.78	63.4	71.9

Data are presented as area under the receiver operating characteristic curve (AUC) with 95% confidence intervals (CI). Optimal cut-off values were determined by receiver operating characteristic (ROC) curve analysis according to the maximum Youden index.

*Age <35 years, hypokalemia, and unilateral adrenal adenoma on CT were analyzed as predefined dichotomous variables; therefore, ROC-derived AUC values are not applicable.

APR, aldosterone-to-potassium ratio; AUC, area under the receiver operating characteristic curve; CI, confidence interval; CT, computed tomography; PAC, plasma aldosterone concentration; ROC, receiver operating characteristic; SIT, saline infusion test.

Bold values indicate statistical significance (p < 0.05).

**Figure 2:**
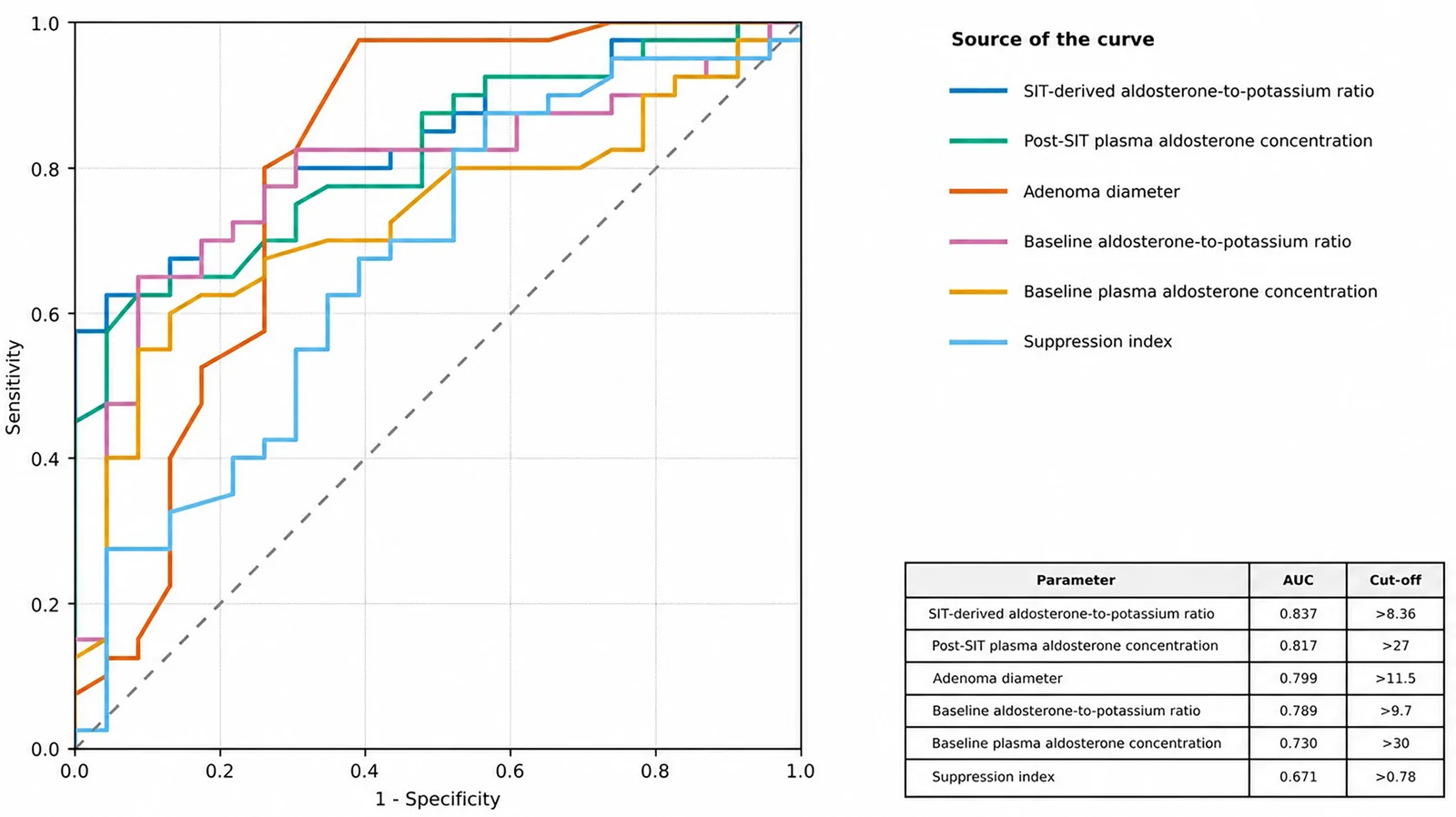
Diagnostic performance of biochemical and radiological parameters for predicting unilateral primary aldosteronism. Receiver operating characteristic (ROC) curves comparing the diagnostic performance of baseline plasma aldosterone concentration, baseline aldosterone-to-potassium ratio, post-saline infusion test (post-SIT) plasma aldosterone concentration, SIT-derived aldosterone-to-potassium ratio, suppression index, and adrenal adenoma diameter for predicting unilateral primary aldosteronism. The corresponding area under the curve (AUC) values and optimal cut-off values determined by the maximum Youden index are summarized in the inset table.

Among the predefined clinical and radiological variables, the presence of a unilateral adrenal adenoma on CT demonstrated the highest diagnostic accuracy, with a sensitivity of 78.0% and a specificity of 87.5%. This was followed by hypokalemia (sensitivity, 63.4%; specificity, 75.0%) and age <35 years (sensitivity, 39.0%; specificity, 90.6%).

Among the continuous variables evaluated by ROC analysis, the SIT-derived APR demonstrated the highest discriminative performance (AUC = 0.837; 95% CI, 0.741–0.933). Using an optimal cut-off value of >8.36, the sensitivity and specificity were 63.4% and 90.6%, respectively. Post-SIT PAC showed similarly high diagnostic performance (AUC = 0.817; 95% CI, 0.716–0.919), with an optimal cut-off value of >27 ng/dL yielding a sensitivity of 63.4% and a specificity of 90.6%. Adrenal adenoma diameter also demonstrated good discriminative ability (AUC = 0.799; 95% CI, 0.668–0.930), with an optimal cut-off value of >11.5 mm providing a sensitivity of 80.0% and a specificity of 73.9%.

Baseline APR (AUC = 0.789), baseline PAC (AUC = 0.730), and the suppression index (AUC = 0.671) also showed significant discriminatory ability for predicting unilateral PA.

Post-SIT PAC and SIT-derived APR were highly correlated (Spearman’s ρ = 0.983, p < 0.001). Their ROC-derived thresholds also showed near-complete agreement in patient classification: 71 of 73 patients (97.3%) were classified concordantly, with only two discordant cases (Cohen’s κ = 0.943, p < 0.001).

Classification performance of the clinical-radiological base model and the expanded models at a predicted-probability threshold of 0.50 is presented in **Supplementary Table S1**. The clinical-radiological base model correctly classified 35 of 41 patients with unilateral PA and classified 4 of 32 patients with bilateral PA as unilateral, corresponding to a sensitivity of 85.4%, specificity of 87.5%, PPV of 89.7%, and NPV of 82.4%. After addition of post-SIT PAC, 39 of 41 patients with unilateral PA were correctly classified, whereas 5 of 32 patients with bilateral PA were classified as unilateral; sensitivity and NPV increased to 95.1% and 93.1%, respectively, while specificity was 84.4% and PPV was 88.6%. The model including SIT-derived APR similarly correctly classified 39 of 41 patients with unilateral PA, while 6 of 32 patients with bilateral PA were classified as unilateral, yielding a sensitivity of 95.1%, specificity of 81.3%, PPV of 86.7%, and NPV of 92.9%. Thus, the higher sensitivity of the SIT-derived models was accompanied by a modest increase in false-positive classification of bilateral PA.

### 3.3 Logistic regression analyses

Univariable logistic regression analyses were performed using the optimal cut-off values identified by ROC analysis for dichotomized biochemical and SIT-derived variables, together with the predefined clinical and radiological variables (**Table 3**).

**Table 3:** Univariable logistic regression analysis of clinical, biochemical, radiological, and SIT-derived predictors of unilateral primary aldosteronism.

Variable	Odds ratio (95% CI)	P value
Clinical and radiological variables		
Age <35 years	6.19 (1.61–23.73)	**0.008**
Hypokalemia (K <3.5 mmol/L)	5.20 (1.87–14.45)	**0.002**
Unilateral adrenal adenoma on CT	24.89 (6.90–89.73)	**<0.001**
Adenoma diameter >11.5 mm	11.33 (3.38–38.04)	**<0.001**
Baseline biochemical variables		
Baseline PAC >30 ng/dL	4.43 (1.63–12.03)	**0.003**
Baseline APR >9.7	8.36 (2.79–25.05)	**<0.001**
SIT-derived variables		
Post-SIT PAC >27 ng/dL	16.76 (4.35–64.50)	**<0.001**
SIT-derived APR >8.36	16.76 (4.35–64.50)	**<0.001**
Suppression index >0.78	4.43 (1.63–12.03)	**0.003**

Odds ratios (ORs) with 95% confidence intervals (CIs) were estimated using univariable logistic regression analysis, with unilateral primary aldosteronism as the dependent variable. Continuous variables were dichotomized according to the optimal cut-off values derived from receiver operating characteristic (ROC) curve analysis using the maximum Youden index.

APR, aldosterone-to-potassium ratio; CI, confidence interval; CT, computed tomography; OR, odds ratio; PAC, plasma aldosterone concentration; PA, primary aldosteronism; ROC, receiver operating characteristic; SIT, saline infusion test.

Bold values indicate statistical significance (p < 0.05).

Among the clinical and radiological variables, the presence of a unilateral adrenal adenoma on CT was the strongest predictor of unilateral PA, conferring an approximately 25-fold higher odds of unilateral disease (OR, 24.89; 95% CI, 6.90–89.73; *p* < 0.001). This was followed by an adrenal adenoma diameter >11.5 mm (OR, 11.33; 95% CI, 3.38–38.04; *p* < 0.001), age <35 years (OR, 6.19; 95% CI, 1.61–23.73; *p* = 0.008), and hypokalemia (OR, 5.20; 95% CI, 1.87–14.45; *p* = 0.002). Among the biochemical variables, a baseline APR >9.7 showed a stronger association with unilateral PA than a baseline PAC >30 ng/dL (OR, 8.36 vs. 4.43). Among the SIT-derived variables, both post-SIT PAC >27 ng/dL and SIT-derived APR >8.36 demonstrated the strongest associations with unilateral PA (both OR, 16.76; 95% CI, 4.35–64.50; *p* < 0.001). A suppression index >0.78 was also significantly associated with unilateral PA (OR, 4.43; 95% CI, 1.63–12.03; *p* = 0.003).

The clinical-radiological base model included age <35 years, hypokalemia, and the presence of a unilateral adrenal adenoma on CT (**Table 4**), and yielded an AUC of 0.936 (95% CI, 0.883–0.990). When each biochemical parameter was added individually to this model, post-SIT PAC >27 ng/dL (adjusted OR, 9.21; 95% CI, 1.29–65.75; *p* = 0.027) and SIT-derived APR >8.36 (adjusted OR, 7.34; 95% CI, 1.04–51.75; *p* = 0.046) remained independently associated with unilateral PA. Likelihood-ratio testing showed significant improvement in model fit after the addition of post-SIT PAC (*p* = 0.014) and SIT-derived APR (*p* = 0.028), whereas the addition of baseline PAC, baseline APR, or the suppression index did not significantly improve model fit. The AUC increased to 0.958 after the addition of post-SIT PAC (ΔAUC = 0.022; 95% CI, −0.010 to 0.054; *p* = 0.185) and to 0.954 after the addition of SIT-derived APR (ΔAUC = 0.017; 95% CI, −0.004 to 0.038; *p* = 0.107); however, neither increase in AUC was statistically significant.

**Table 4:** Comparative performance of biochemical and SIT-derived parameters added to the common clinical-radiological model.

Model	Adjusted OR (95% CI)	P value	AUC (95% CI)	ΔAUC vs base (95% CI)	Paired AUC comparison, P	LR test, P	Optimism-corrected AUC
Clinical-radiological base model	—	—	0.936 (0.883–0.990)	—	—	—	0.926
+ Baseline PAC >30 ng/dL	0.67 (0.15–3.03)	0.604	0.939 (0.886–0.992)	+0.003 (−0.007 to 0.013)	0.606	0.604	0.925
+ Baseline APR >9.7	3.83 (0.73–20.05)	0.112	0.946 (0.897–0.996)	+0.010 (−0.011 to 0.030)	0.342	0.098	0.937
+ Suppression index >0.78	0.50 (0.11–2.31)	0.374	0.941 (0.889–0.993)	+0.005 (−0.009 to 0.019)	0.495	0.372	0.928
+ Post-SIT PAC >27 ng/dL	9.21 (1.29–65.75)	0.027	0.958 (0.913–1.000)	+0.022 (−0.010 to 0.054)	0.185	**0.014**	0.945
+ SIT-derived APR >8.36	7.34 (1.04–51.75)	0.046	0.954 (0.908–0.999)	+0.017 (−0.004 to 0.038)	0.107	**0.028**	0.939

Adjusted odds ratios (ORs) with 95% confidence intervals (CIs) were estimated using multivariable logistic regression analysis, with unilateral primary aldosteronism as the dependent variable. The clinical-radiological base model included age <35 years, hypokalemia (serum potassium <3.5 mmol/L), and unilateral adrenal adenoma on computed tomography. Each biochemical or SIT-derived parameter was added separately to the base model. Differences in discrimination were assessed using paired comparisons of the area under the receiver operating characteristic curves (ΔAUC), and nested models were compared using likelihood-ratio (LR) tests. Internal validation was performed using 2,000 bootstrap resamples to estimate optimism-corrected AUCs.

APR, aldosterone-to-potassium ratio; AUC, area under the receiver operating characteristic curve; CI, confidence interval; CT, computed tomography; LR, likelihood-ratio; OR, odds ratio; PAC, plasma aldosterone concentration; PA, primary aldosteronism; SIT, saline infusion test.

Bold values indicate statistical significance (p < 0.05).

Internal validation using 2,000 bootstrap resamples showed limited optimism in model discrimination. The optimism-corrected AUCs were 0.926 for the clinical-radiological base model, 0.945 for the model including post-SIT PAC, and 0.939 for the model including SIT-derived APR. In an additional sensitivity analysis in which biomarker cut-off selection using the maximum Youden index was repeated within each bootstrap resample, estimated optimism remained modest (approximately 0.016–0.020).

Calibration assessment showed bootstrap-corrected Brier scores of 0.117, 0.097, and 0.102 for the clinical-radiological base model, the model including post-SIT PAC, and the model including SIT-derived APR, respectively. The corresponding bootstrap-corrected calibration slopes were 0.67, 0.65, and 0.63. Calibration plots showed generally close agreement between predicted and observed probabilities across the three models (**Supplementary Figure S1**).

## 4 Discussion

Accurate identification of unilateral PA is essential for selecting patients who are most likely to achieve biochemical remission and clinical benefit from adrenalectomy. Current clinical practice guidelines recommend AVS for subtype classification in patients with PA who are candidates for adrenalectomy, while recognizing that AVS may be considered unnecessary in selected young patients with a high probability of unilateral disease, characterized by features such as spontaneous hypokalemia, marked PA, and a unilateral adrenal adenoma on CT (9). Although marked PA is exemplified by biochemical features including elevated PAC and suppressed renin, the relative diagnostic contribution of different biochemical parameters to the identification of unilateral disease remains uncertain. In the present study, we evaluated the diagnostic value of biochemical parameters obtained before and after the SIT in patients with AVS-confirmed PA subtypes. In our cohort, post-SIT PAC and SIT-derived APR showed the highest numerical AUCs among the evaluated biochemical parameters and remained associated with unilateral PA when individually added to the common clinical-radiological framework. However, these two parameters were very strongly correlated and showed near-complete agreement in patient classification, indicating that they largely captured overlapping diagnostic information.

The SIT is routinely used as a confirmatory test for PA and has recently attracted increasing interest as a potential tool for distinguishing unilateral from bilateral disease. Accordingly, several SIT-derived biochemical parameters, including post-SIT PAC, and the aldosterone suppression rate, have been investigated for their diagnostic performance in PA subtyping (10, 12–16). Among these, post-SIT PAC is the most extensively studied SIT-derived biomarker. Previous studies have consistently demonstrated its value in differentiating unilateral from bilateral PA. Nagano et al. reported that a post-SIT PAC <246 pmol/L predicted bilateral PA with a sensitivity of 80.3% and a specificity of 86.2% (10). Similarly, Hashimura et al. found that a post-SIT PAC <300 pmol/L predicted bilateral disease with a sensitivity of 76.2% and a specificity of 75.8% (16). Consistent with these findings, post-SIT PAC demonstrated strong discriminative performance for predicting unilateral PA in our cohort (AUC = 0.817). A cut-off value of >27 ng/dL yielded a sensitivity of 63.4% and a specificity of 90.6%. These findings support the potential utility of post-SIT PAC for PA subtyping. When post-SIT PAC was added to the common clinical-radiological framework, it remained independently associated with unilateral PA and significantly improved model fit. However, the corresponding increase in AUC was modest and did not reach statistical significance, indicating that its additional discriminatory contribution should be interpreted cautiously.

The APR has been investigated as a potential biochemical parameter for PA subtyping because it incorporates both aldosterone concentration and serum potassium. Puar et al. demonstrated that the APR, calculated using the highest plasma aldosterone concentration and the lowest serum potassium concentration, was useful for differentiating unilateral from bilateral PA. An APR >15 predicted unilateral disease with a probability of 91.7%, whereas an APR <5 predicted bilateral disease with a probability of 76.7% (17). Subsequently, Barlas et al. incorporated APR into a clinical prediction model and reported that an APR >12 improved the prediction of unilateral PA (18). Consistent with these findings, baseline APR demonstrated good diagnostic performance in our cohort (AUC = 0.789), with a cut-off value of >9.7 yielding a sensitivity of 65.9% and a specificity of 81.2%. However, previous studies have not clearly established the additional diagnostic information provided by baseline APR when evaluated within the same clinical-radiological framework as SIT-derived biochemical parameters. To our knowledge, this is the first study to address this question. Although baseline APR showed favorable individual diagnostic performance, it was not independently associated with unilateral PA after adjustment for the clinical-radiological framework used in our analysis. Given previous evidence that post-SIT PAC may show greater discriminatory performance than baseline PAC for PA subtyping, we evaluated whether combining post-SIT PAC with the pre-intervention minimum serum potassium concentration as SIT-derived APR might provide additional diagnostic information. SIT-derived APR showed a numerically higher AUC than baseline APR (0.837 vs. 0.789) and remained associated with unilateral PA when added to the common clinical-radiological framework. However, SIT-derived APR was very strongly correlated with post-SIT PAC (Spearman’s ρ = 0.983), and their ROC-derived thresholds classified 71 of 73 patients (97.3%) concordantly. These findings indicate that SIT-derived APR and post-SIT PAC largely capture overlapping diagnostic information and do not support superiority of SIT-derived APR or a substantial diagnostic contribution beyond that provided by post-SIT PAC.

Biochemical parameters reflecting the degree of aldosterone suppression following the SIT have recently gained attention as potential biomarkers for PA subtyping. Nagano et al. were the first to evaluate the aldosterone suppression rate using ROC analysis and reported that an aldosterone suppression rate >33.8% predicted bilateral PA with a sensitivity of 87.2% (10). Subsequently, Barlas et al. incorporated the same parameter into a clinical prediction model and demonstrated that a suppression rate >43.5% contributed to subtype discrimination (18). More recently, Mansour et al. evaluated the ratio of baseline to post-SIT PAC within an integrated prediction model and found that this parameter differed significantly between unilateral and bilateral disease (19). In the present study, we evaluated the suppression index (post-SIT PAC/baseline PAC), a parameter reflecting a similar physiological concept. The suppression index demonstrated significant discriminative performance for predicting unilateral PA (AUC = 0.671), and a cut-off value of >0.78 was significantly associated with unilateral disease in univariable analysis (OR, 4.43; 95% CI, 1.63–12.03). However, the suppression index was not independently associated with unilateral PA when added to the common clinical-radiological framework. These findings suggest that although the suppression index may provide discriminatory information for PA subtyping, its additional diagnostic contribution beyond the clinical and radiological features included in our model may be limited.

One of the notable findings of our study was that post-SIT PAC showed numerically higher discriminative performance than baseline PAC for predicting unilateral PA. Although baseline PAC was associated with unilateral PA in univariable analysis, this association did not persist when it was added to the common clinical-radiological framework. In contrast, post-SIT PAC remained associated with unilateral PA when added to the same framework and significantly improved model fit. However, the increase in AUC was modest and did not reach statistical significance, indicating that the additional discriminatory contribution of post-SIT PAC beyond the clinical and radiological features should be interpreted cautiously. This difference between post-SIT and baseline PAC may be partly explained by the underlying pathophysiology of PA. Under physiological conditions, saline loading suppresses the renin–angiotensin–aldosterone system, resulting in a reduction in aldosterone secretion. In bilateral PA, aldosterone secretion generally retains partial responsiveness to volume expansion, whereas in unilateral PA, aldosterone secretion may exhibit a greater degree of autonomy and remain inadequately suppressed despite saline loading (7, 20, 21). Consequently, post-SIT PAC may reflect not only the degree of aldosterone excess but also the persistence of aldosterone secretion during saline loading. This may partly explain the numerically higher discriminative performance of post-SIT PAC compared with baseline PAC observed in our cohort.

Our study has several limitations. First, the relatively small sample size and the retrospective single-center design may have introduced selection bias and may limit the generalizability of our findings. In addition, the SIT was performed exclusively using the recumbent protocol. Seated SIT has been reported to have greater sensitivity, particularly in patients with milder PA phenotypes. Therefore, the exclusive use of the recumbent protocol may have resulted in underrepresentation of patients with less pronounced biochemical disease and selection toward a more florid PA phenotype. This may have influenced the observed diagnostic performance of the post-SIT biochemical parameters. Similarly, the use of an immunoassay for PAC measurement in our study should be considered. Immunoassays may have lower analytical accuracy than liquid chromatography–tandem mass spectrometry (LC–MS/MS), particularly at low aldosterone concentrations, which may have affected post-SIT PAC measurements and the derived cut-off values. Second, the ROC-derived cut-off values were determined in the same cohort in which their subsequent model performance was evaluated, which may have introduced optimism and increased the risk of overfitting. Although bootstrap internal validation was performed to assess this issue, internal validation does not replace validation in an independent external cohort. Moreover, although the addition of post-SIT PAC and SIT-derived APR to the common clinical-radiological framework significantly improved model fit, the corresponding increases in AUC were modest and did not reach statistical significance. Therefore, the additional discriminatory information provided by these parameters beyond the clinical and radiological features should be interpreted cautiously. Considering also the inter-laboratory differences in aldosterone measurement methods, the derived cut-off values and model performance require external validation in larger independent cohorts, preferably in multicenter prospective studies using different SIT protocols and aldosterone measurement methods. Third, PA subtype classification was based on AVS as the functional reference standard. Although AVS is the established reference method for subtype classification in patients with PA who are candidates for adrenalectomy, postoperative biochemical outcomes could have provided additional confirmation of unilateral disease. Of the 41 patients classified as having unilateral PA, 33 underwent adrenalectomy; however, paired postoperative aldosterone and renin measurements at standardized follow-up were not systematically available. Therefore, postoperative biochemical remission could not be reliably assessed in a standardized manner. Moreover, the absence of an independent and universally accepted reference standard for bilateral PA represents an inherent limitation of studies evaluating PA subtyping. Finally, the model-based classification results should be interpreted cautiously in terms of their potential clinical application. The increased sensitivity observed after incorporating post-SIT biochemical parameters was accompanied by a modest increase in the false-positive classification of bilateral PA as unilateral disease. Therefore, the models evaluated in this study should not be interpreted as validated clinical decision rules for omitting AVS or directing patients to adrenalectomy without AVS. Despite these limitations, our study has several strengths. AVS-based subtype classification and the systematic comparison of baseline and SIT-derived biochemical parameters within the same common clinical-radiological framework allowed their additional diagnostic information to be evaluated on a comparable basis. Furthermore, the model comparisons were complemented by internal validation and calibration analyses. Nevertheless, external validation in independent populations is required before these findings can be translated into clinical practice.

## 5 Conclusion

In conclusion, this study evaluated the potential diagnostic value of biochemical parameters derived from the saline infusion test for PA subtyping. Our findings suggest that post-SIT PAC shows useful discriminative performance for predicting unilateral PA and may contribute to subtype assessment when considered alongside clinical and radiological features. Further validation in larger independent multicenter prospective studies is needed to establish its potential role in clinical practice.

## Data Availability

The raw data supporting the conclusions of this article will be made available by the authors, without undue reservation.

## Supplementary material

Supplementary Figure 1 Calibration plots of the models for predicting unilateral primary aldosteronism. Calibration plots comparing predicted probabilities with observed proportions of unilateral primary aldosteronism for the clinical-radiological base model, the base model including post-SIT PAC, and the base model including SIT-derived APR. Observed proportions were estimated across five groups based on quantiles of predicted probability. The dashed 45° line represents perfect calibration.
