# Supplementary material for: Diagnostic value of saline infusion test–derived biochemical parameters for primary aldosteronism subtyping
Source: Front Endocrinol (Lausanne). 2026 Oct 6;17:1923356. doi: 10.3389/fendo.2026.1923356 (PMC13680153; doi:10.3389/fendo.2026.1923356)
Supplement: Supplementary file 1 [file Table1.docx]

**Supplementary Table S1. Classification performance of the clinical-radiological base model and models incorporating biochemical and SIT-derived parameters for unilateral primary aldosteronism**

| **Model** | **TP, n** | **FP, n** | **TN, n** | **FN, n** | **Sensitivity (%)** | **Specificity (%)** | **PPV (%)** | **NPV (%)** |
| --- | --- | --- | --- | --- | --- | --- | --- | --- |
| Clinical-radiological base model | 35 | 4 | 28 | 6 | 85.4 | 87.5 | 89.7 | 82.4 |
| Base model + baseline PAC >30 ng/dL | 36 | 5 | 27 | 5 | 87.8 | 84.4 | 87.8 | 84.4 |
| Base model + baseline APR >9.7 | 36 | 5 | 27 | 5 | 87.8 | 84.4 | 87.8 | 84.4 |
| Base model + suppression index >0.78 | 36 | 6 | 26 | 5 | 87.8 | 81.3 | 85.7 | 83.9 |
| Base model + post-SIT PAC >27 ng/dL | 39 | 5 | 27 | 2 | 95.1 | 84.4 | 88.6 | 93.1 |
| Base model + SIT-derived APR >8.36 | 39 | 6 | 26 | 2 | 95.1 | 81.3 | 86.7 | 92.9 |
| Classification performance was evaluated using a predicted-probability threshold of 0.50, with unilateral PA considered the positive outcome. The clinical-radiological base model included age <35 years, hypokalemia (serum potassium <3.5 mmol/L), and the presence of a unilateral adrenal adenoma on CT. Each biochemical or SIT-derived parameter was added individually to the base model. TP denotes patients with unilateral PA correctly classified as unilateral; FP, patients with bilateral PA classified as unilateral; TN, patients with bilateral PA correctly classified as bilateral; and FN, patients with unilateral PA classified as bilateral. PPV and NPV were calculated from the corresponding classification tables.  **Abbreviations:** APR, aldosterone-to-potassium ratio; CT, computed tomography; FN, false negative; FP, false positive; NPV, negative predictive value; PA, primary aldosteronism; PAC, plasma aldosterone concentration; PPV, positive predictive value; SIT, saline infusion test; TN, true negative; TP, true positive. | | | | | | | | |
